# Supplementary material for: FliO is an evolutionarily conserved yet diversified core component of the bacterial flagellar type III secretion system
Source: bioRxiv. 2025 May 6:2025.05.06.652439. Preprint. [Version 1] doi: 10.1101/2025.05.06.652439 (PMC12248157; doi:10.1101/2025.05.06.652439)
Supplement: Supplement 1 [file media-1.pdf]

Supplementary Information

FliO is an evolutionarily conserved yet diversified core component of the bacterial flagellar type III secretion system

Ekaterina P. Andrianova, Amanda L. Dobbins, Marc Erhardt, David R. Hendrixson, Igor B. Zhulin

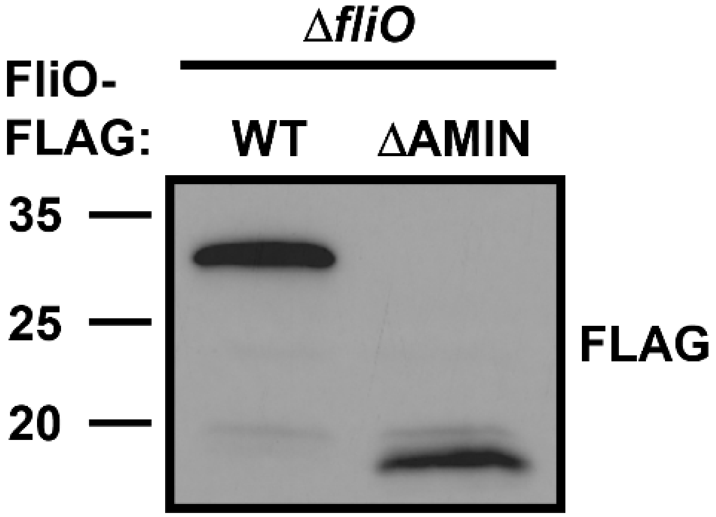

Supplementary Fig. 1: Immunoblot analysis of immunoprecipitated WT FliO-FLAG and FliO$_{\Delta AMIN}$-FLAG. *C. jejuni* Δ*fliO* was complemented in the *rrsC* locus with the native *fliNO* promoter expressing *fliO-FLAG* or *fliO*$_{\Delta AMIN}$-*FLAG*. Strains were grown and then diluted to OD_{600} of 0.8. Proteins were immunoprecipitated with a-FLAG resin and, after washing, resuspended in equal volume of 1X SDS-PAGE loading buffer. Equal volumes of samples were examined. a-FLAG antibody was used to detect FliO-FLAG and FliO$_{\Delta AMIN}$-FLAG.

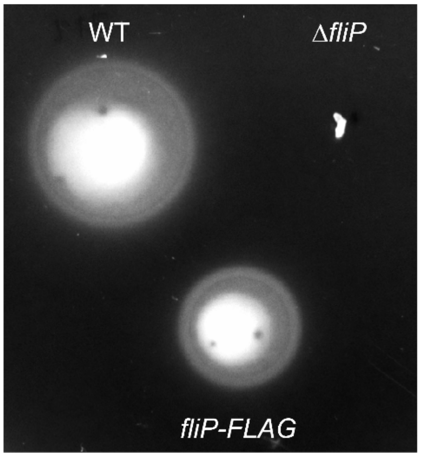

Supplementary Fig. 2: Characterization of swimming motility of *C. jejuni* strains producing FliP-FLAG. (A) Representative image of the motility phenotypes of WT *C. jejuni* (top left), Δ*fliP* (top right), and *fliP-FLAG* (bottom). Strains were diluted to an OD_{600} of 0.8 and then stabbed into MH motility agar containing 0.4% agar. Motility was analyzed after 30 h of incubation at 37 °C in microaerobic conditions.

Supplementary Table 1: Bacterial strains used in this study

Strain	Genotype	Source/Reference
***E. coli* strains**		
DH5α	*E. coli supE44* Δ*lacU169* (φ80*lacZ*ΔM15) *hsdR17 recA1 endA1 gyrA96 thi-1 relA1*	Invitrogen
DH5α / pRK212.1	DH5α with conjugation transfer element	1
BL21(DE3)	*E. coli fhuA2 [lon] ompT gal (λ DE3) [dcm]* Δ*hsdS λ DE3 = λ sBamHIo* Δ*EcoRI-B int::(lacI::PlacUV5::T7 gene1) i21* Δ*nin5*	New England Labs
***Campylobacter jejuni* strains**		
DRH212	81-176 *rpsL*^{Sm}	2
DRH706	81-176 *rpsL*^{Sm} *fliP::cat-rpsL*	3
DRH755	81-176 *rpsL*^{Sm} Δ*fliR*	3
DRH964	81-176 *rpsL*^{Sm} Δ*flhA*	3
DRH1065	81-176 *rpsL*^{Sm} Δ*fliP*	3
DRH8073	81-176 *rpsL*^{Sm} *fliO::cat-rpsL*	This study
DRH8113	81-176 *rpsL*^{Sm} Δ*fliO*	This study
DAR101	81-176 *rpsL*^{Sm} Δ*fliQ*	4
SMS508	81-176 *rpsL*^{Sm} *fliQ::cat-rpsL*	4
SNJ471	81-176 *rpsL*^{Sm} Δ*flhB*	5
ALD962	81-176 *rpsL*^{Sm} Δ*fliO fliR::kan-rpsL*	This study
ALD963	81-176 *rpsL*^{Sm} Δ*fliO fliP::cat-rpsL*	This study
ALD965	81-176 *rpsL*^{Sm} Δ*fliO fliQ::cat-rpsL*	This study
ALD1050	81-176 *rpsL*^{Sm} Δ*fliO rrsC::kan-*P_{fliNO}*-fliO-FLAG*	This study
ALD1081	81-176 *rpsL*^{Sm} Δ*fliO rrsC::kan-*P_{fliNO}*-*$fliO_{\Delta AMIN}$*-FLAG*	This study
ALD1106	81-176 *rpsL*^{Sm} Δ*fliO* $fliP_{P22\text{-}FLAG\text{-}T23}$	This study
ALD1130	81-176 *rpsL*^{Sm} $fliP_{P22\text{-}FLAG\text{-}T23}$	This study

Supplementary Table 2: Plasmids used in this study

Plasmid	Genotype	Source/Reference
pUC19	Amp^R; general cloning vector	New England Biolabs
pBR322	Amp^R; general cloning vector	New England Biolabs
pILL600	Kan^R; source of *kan* cassette	6
pDRH265	pUC19 containing *cat-rpsL*	3
pDRH643	pUC19 containing *fliR*::*cat-rpsL*	3
pDRH645	pUC19 containing *fliP*::*cat-rpsL*	3
pDRH2455	pUC19 containing *fliO* with 0.7 kb upstream and downstream cloned into the BamHI site	This study
pDRH2547	pUC19 with DNA fragment to create an in-frame deletion of codons 2 to 263 of *fliO* cloned into the BamHI site	This study
pDRH2559	pUC19 containing *fliO* with 0.7 kb of upstream and downstream sequence cloned into the BamHI site; contains T410G mutation to create an StuI site in *fliO*	This study
pDRH2568	SmaI-digested *cat-rpsL* cassette cloned into the StuI site of *fliO* in pDRH2559	This study
pDRH7574	pBR322 with a 2.6 kb DNA fragment containing a portion of the coding sequence of *rrsC* through a portion of *rrlC* was cloned into the EcoRI site	This study
pDRH7746	*kan* cassette from pILL600 cloned into the XbaI site of the *rrsC* locus of pDRH7574	This study
pSMS469	pUC19 containing *fliQ*::*cat-rpsL*	4
pALD1047	*fliNO* promoter from 120 bp up to the *fliN* start codon fused to the start codon of the *fliO* coding sequence with DNA encoding a C-terminal FLAG tag cloned into the XbaI site of pDRH7746	This study
pALD1059	*fliNO* promoter from 120 bp up to the *fliN* start codon fused to the start codon of the *fliO* coding sequence lacking the predicted AMIN domain (codons 21-118) with	This study

	DNA encoding a C-terminal FLAG tag cloned into the XbaI site of pDRH7746	
pALD1062	*fliP* locus with 0.7 kb of upstream and downstream sequence with DNA encoding a FLAG tag between codons 22 and 23 to encode $FliP_{P22\text{-}FLAG\text{-}T23}$ cloned into the EcoRI site of pUC19	This study

Supplementary Table 3: Identification by mass spectrometry of fT3SS components isolated from total membrane fractions of WT *C. jejuni* and the Δ*fliO* mutant.

Gene ID	Protein Name	WT/Δ*fliO* Abundance*
CJJ81176_0837	FliP	0.79
CJJ81176_0357	FlhB	2.26
CJJ81176_0890	FlhA	0.94
CJJ81176_0340	FliF	1.13
CJJ81176_0341	FliG	1.01
CJJ81176_0502	SecG	0.99

*The ratio of the abundance of each protein in WT *C. jejuni* and *C. jejuni* Δ*fliO* was calculated. FliR and FliQ were not detected by mass spectrometry. SecG is a non-flagellar membrane protein that served as a control for a protein that should be at similar levels in the *C. jejuni* strains.

REFERENCES

1. Figurski, D. H. & Helinski, D. R. Replication of an origin-containing derivative of plasmid RK2 dependent on a plasmid function provided in *trans*. *Proc. Natl. Acad. Sci. USA* **76**, 1648–1652 (1979).
2. Hendrixson, D. R., Akerley, B. J. & DiRita, V. J. Transposon mutagenesis of *Campylobacter jejuni* identifies a bipartite energy taxis system required for motility. *Mol. Microbiol.* **40**, 214–224 (2001).
3. Hendrixson, D. R. & DiRita, V. J. Transcription of σ^{54}-dependent but not σ^{28}-dependent flagellar genes in *Campylobacter jejuni* is associated with formation of the flagellar secretory apparatus. *Mol. Microbiol.* **50**, 687–702 (2003).
4. Balaban, M. & Hendrixson, D. R. Polar Flagellar Biosynthesis and a Regulator of Flagellar Number Influence Spatial Parameters of Cell Division in *Campylobacter jejuni*. *PLoS Pathog.* **7**, e1002420 (2011).
5. Joslin, S. N. & Hendrixson, D. R. Activation of the *Campylobacter jejuni* FlgSR Two-Component System Is Linked to the Flagellar Export Apparatus. *J. Bacteriol.* **191**, 2656–2667 (2009).
6. Labigne-Roussel, A., Courcoux, P. & Tompkins, L. Gene disruption and replacement as a feasible approach for mutagenesis of Campylobacter jejuni. *J. Bacteriol.* **170**, 1704–1708 (1988).
